# Evolution of Shear Surface Morphology of Jointed Rock Masses Based on Gaussian Filtering Method under Freeze-Thaw Cycles

**DOI:** 10.3390/ma15124228

**Published:** 2022-06-15

**Authors:** Daxing Lei, Yifan Chen, Hang Lin, Chunshun Zhang, Zhigang Lu, Guangli Wang, Yaoping Zhang

**Affiliations:** 1 School of Resources and Architectural Engineering, Gannan University of Science and Technology, Ganzhou 341000, China; daxinglei17@163.com (D.L.); lufangang9901@163.com (Z.L.); guangliwang95@163.com (G.W.); yaopingzhang74@163.com (Y.Z.); 2 Key Laboratory of Mine Geological Disaster Prevention and Control and Ecological Restoration, Ganzhou 341000, China; 3 School of Resources and Safety Engineering, Central South University, Changsha 410083, China; linhangabc@126.com; 4 Department of Civil Engineering, Monash University, Clayton, VIC 3800, Australia

**Keywords:** freeze-thaw cycles, direct shear experiment, Gaussian filtering, shear surface morphology, large-scale waviness surface, small-scale unevenness surface

## Abstract

This study aims to quantify the shear surface morphology of jointed rock and its evolution under shearing, cyclic freezing, and thawing using the Gaussian filtering method. Gaussian filtering method enables the construction of the (large-scale) waviness surface and the (small-scale) unevenness surface of a digitized surface (created by laser scanning). Both waviness and unevenness surfaces are then quantified by roughness coefficient ratio (*S*) and degradation degrees of the waviness surface (*D_w_*) and unevenness surface (*D_r_*). These (microscopic) morphological parameters (*S*, *D_w_* and *D_r_*) are subsequently used to explain the development of the (macroscopic) shear strength of the jointed rocks on direct shear tests. The results indicate that compared with fresh jointed rocks, the freezing and thawing causes the potential shear surface asperities to be easier to damage and fail under shear load. Such damage is well represented by the significant decrease in *D_w_* and *D_r_*. On the other hand, with the increase of the freeze-thaw cycle (*N*), *D_w_* increases while *D_r_* reaches the maximum at an early stage of the cycle, where *D_r_* > *D_w_*. This difference reveals the underlying shear mechanism microscopically; that is, in the initial stage, the shear surface morphology is mainly dominated by the unevenness surface *D_r_*, and then it is controlled by the waviness surface *D_w_* during the freeze-thaw cycle.

## 1. Introduction

Non-persistent joints are widely encountered in rock masses in cold regions, and the freeze-thaw cycle effect shows significant deterioration characteristics on the mechanical properties of rock masses, which will cause serious safety hazards to rock mass engineering in cold regions [1,2,3,4,5,6]. Recent work reveals that under the action of freeze-thaw cycling, non-persistent joints are damaged to varying degrees, and the damage propagates to the inside of the rock mass, causing new cracks on both sides of the joint [7,8]. Cracks caused by the joint are prone to spread in the rock mass under shearing to form a shear (failure) surface [9,10,11,12,13]. Especially under the freeze-thaw cycle in cold areas, the strength of the rock mass will be significantly reduced, which is reflected in the joint-related shear surface morphology and its evolution [14,15].

The joint-related shear surface morphology is generally composed of (large-scale) first-order asperities and (small-scale) second-order asperities [16,17]. From the point of rock mechanics, the first-order asperities are denoted as the (large-scale) waviness, and the second-order asperities are known as the (small-scale) unevenness [18,19]. The degrees of the waviness and unevenness would be significantly degraded due to the freeze-thaw damage, resulting in the change of the surface morphology, deteriorating the mechanical properties of the shear surface. 

Existing research rarely investigates the evolution of joint-related shear surface morphology and its impact on the shear strength of the jointed rock; instead, there are a few studies on the influences of joint distributions and evolution of frost-heave pressures on the rock strength. Although not directly relevant, the latter studies provide some indirect basis to understand the role of freeze-thaw cycles on the mechanical properties of rock masses. For example, Ghobadi et al. [20] discussed the variations of physical and mechanical parameters of rock under freeze-thaw cycles; Wang et al. [21] analyzed the relationship between rock mechanical parameters and freeze-thaw damage. Among various mechanical parameters, the shear characteristics of the jointed rock mass under freeze-thaw cycles have attracted special and broad attention. For instance, Krautblatter et al. [22] described the influence of an ice-containing structural surface on slope stability and discussed the variation of shear resistance caused by ice slag. Mu et al. [23] proposed the influence trend and damage model of freeze-thaw cycles on the shear strength of the rock masses with three types of joints based on the shear tests. The research of Davies et al. [24] showed the difference between the shear test curve of frozen jointed rock and that of natural jointed rock. Lei et al. [25] explored the changes in shear strength of jointed rock masses with different persistence rates after multiple freeze-thaw cycles. The research results principally reveal the degradation mechanism of the shear strength of jointed rock mass subjected to different freezing-thawing weathering cycles, and the reduction variations of shear strength parameters. However, there is no relation between the weakening of joint shear strength and the change of meso-roughness of the shear plane. Wang et al. [26] investigated the strength loss and failure mechanism of the mortar-rock bonding interface caused by freeze-thaw cycles. However, as earlier mentioned, all the above and other current research lacks the consideration on the characteristics of shear surface morphology of the jointed rock mass, let alone the insight into the morphological changes in (large-scale) surface waviness and (small-scale) unevenness.

This study aims to quantify the shear surface morphology and its evolution under shearing, cyclic freezing, and thawing using the Gaussian filtering method. First, the freeze-thaw cycle experiment and multiple shear tests were successively performed on the rock-like joints. Then, the 3D topography data of the shear surfaces of jointed rock masses before and after each shear test were obtained using a 3D high-precision non-contact laser profiler. After that, based on the Gaussian filter analysis method, the scanned shear surface was digitized into the (large-scale) waviness surface and the (small-scale) unevenness surface and the characteristics of both digitized surfaces after shearing were investigated separately. Finally, the parameters that characterize the wear degree of the shear surface under freeze-thaw cycles were proposed, which provides a new analysis method for the study on the damage to jointed rock shear strength by freeze-thaw cycling.

## 2. Experiment Overviews

### 2.1. Preparation of Jointed Rock Specimens

It is well known that creating a regular joint in a natural rock specimen is challenging. In contrast, it is much more straightforward to prefabricate the regular joint in a rock-like material (with a proper proportion of cement and mortar to ensure similar strength to the natural rock), which has become a common practice in rock testing. Therefore, we also adopted the general experimental procedure to prepare the artificial-jointed sandstone specimens for the current study [27]. 

**Figure 1:**
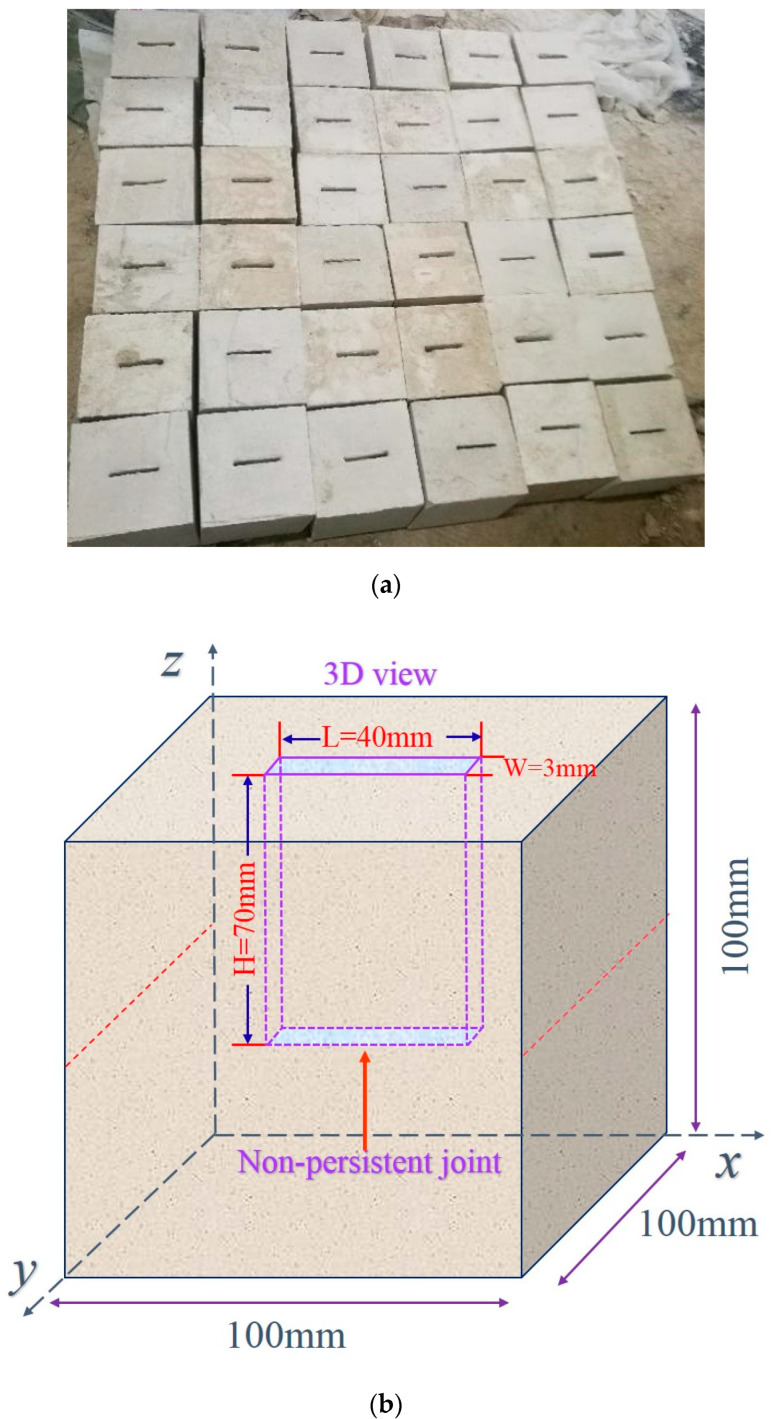
Non-persistent jointed rock specimen. (**a**) Jointed rock specimens. (**b**) Schematic diagram of the jointed rock specimen.

In order to make specimens with different sizes of joints, similar experimental methods were used in this experiment. In the initial stage of the experiment, acrylic plates were inserted into cement mortar in advance. After the mortar was completely solidified and maintained in a constant temperature and humidity environment, rock specimens containing different joints were completed. The size of a rock specimen can be seen from Figure 1, with the non-persistent joint of 3 mm (thickness) × 40 mm (width) × 70 mm (length). With this regular joint, the frost heaving force generated by the joint can be easily calculated. Next, we first started the freeze-thaw cycle test and then conducted the direct shear test. For more information about the cement-mortar ratio, technical detail, test procedures of the artificial sandstone specimens, freeze-thaw test process, and shear test details, please refer to our recent work of Lei et al. [25].

### 2.2. Experimental Equipment and Scheme

**Figure 2:**
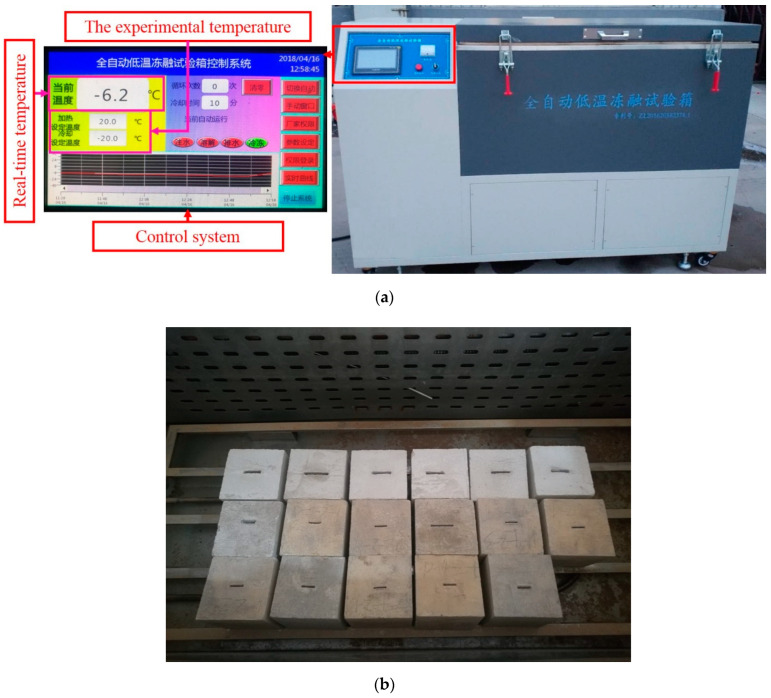
Freeze-thaw cycle experiment equipment and process. (**a**) TDS-300 automatic low-temperature freeze-thaw experiment container. (**b**) Specimens during the freeze-thaw experiment.

**Figure 3:**
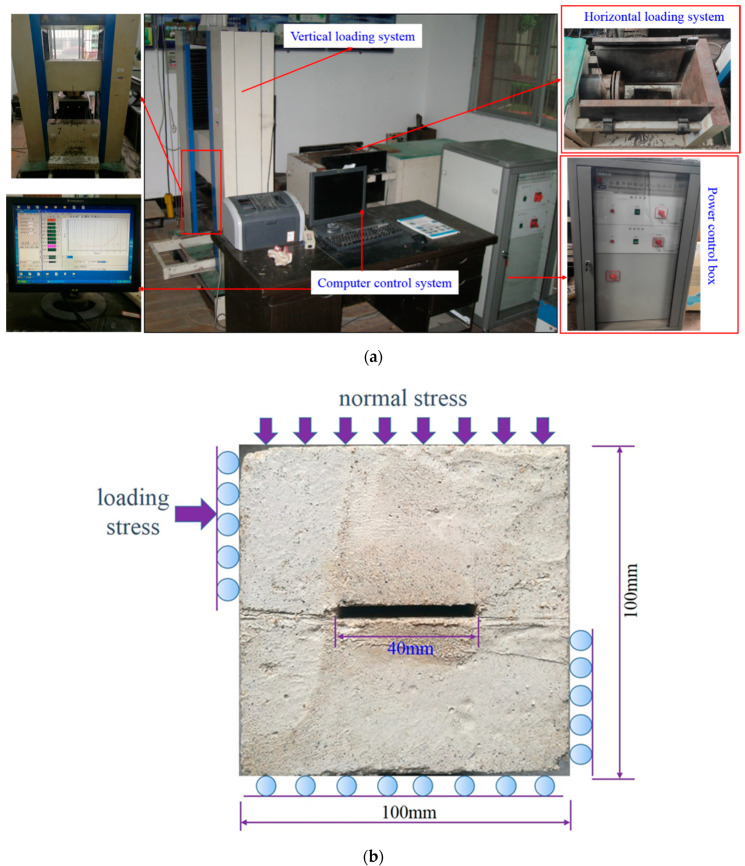
Rock Shear Tester and Schematic diagram of shear principle. (**a**) Shear testing equipment. (**b**) Schematic diagram of shearing of joint specimen.

**Figure 4:**
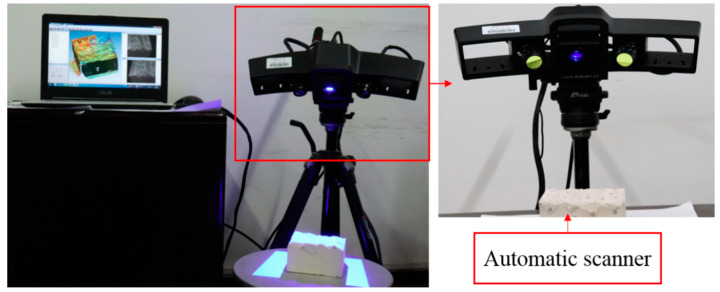
HL-3DS camera-type 3D scanner.

This experimental investigation is mainly divided into freeze-thaw cycling, shearing, and shear surface scanning. The corresponding equipment includes the following: (1) The automatic low-temperature freeze-thaw test machine (CMEC Sanyou group, Tianjin, China) is used in the freeze-thaw experiment (see Figure 2); (2) The shearing equipment and specimen shear diagram are shown in Figure 3; and (3) The topography of the jointed rock mass shear surface is scanned using the HL-3DS camera-type three-dimensional scanner, as shown in Figure 4. 

With the above three test facilities, the experimental scheme is briefly introduced below.

(1) In the conventional direct shear test, some joint samples were subjected to three cyclic shear tests and were grouped according to the number of freeze-thaw cycles. In the test process, the joint shear surface was scanned after each shear test. Then, after the scanning was completed, the upper and lower parts of the sample were placed in coincidence. Before the second shear, the joint sample was closed to ensure that the upper and lower sides of the joint were in a state of joint bonding.
(2) After each shearing experiment, the shear surface was scanned at the point spacing of 0.01 mm to obtain the morphology of the jointed rock mass shear surface. Owing to the non-contact laser scanning technique, the acquisition process of elevation data will not damage the shear surface. Then, combined with the supporting data analysis software TalyMap, the morphology data can be processed and analyzed. The scanning measurement follows the principle of triangular optics; the whole process is to convert the asperities data of the shear surface topography into a current signal through the photoelectric conversion system. Then, the current signal after magnification and analogue-to-digital conversion, the reading, storage, processing, and three-dimensional visualization of the shear surface topography can be realized. In doing so, the obtained surface morphology parameters can accurately represent the roughness of the shear surface.

## 3. Gaussian Filtering Principle

### 3.1. Gaussian Filtering on the Shear Surface

The shear surface of the rock is exceptionally irregular, which contains a variety of frequency components. Typically, a low-frequency component corresponds to a (large-scale) surface waviness that is considered as the first-order asperity. As a comparison, a high-frequency component corresponds to a (small-scale) surface unevenness that is considered as the second-order asperity. The superposition of the low-frequency waviness and high-frequency unevenness consists of the full surface morphology. 

A straightforward way to extract the morphology of the low-frequency waviness is to use the Gaussian filtering method. Complete understanding of the method is a challenge, and beyond the scope of this paper, so only a brief idea is elaborated as follows. 

The Gaussian filtering method is essentially a low-pass filter for the frequency domain. The range of filtering is related to a Gaussian weight function [28],

(1) $$f(x)=\frac{1}{\alpha \lambda_{c}}\exp\left[-\pi {\left(\frac{x}{\alpha \lambda_{c}}\right)}^{2}\right]$$

and its transformation function Equation (1) after Fourier transform. In doing so, the sinusoidal waves used for the shear surface in scanning would be converted into the low-frequency morphological signal indicating the surface waviness condition. Then, the surface unevenness condition is the frequency difference between the shear surface and the obtained surface waviness.

### 3.2. Surface Morphology Analysis after Gaussian Filtering

**Figure 5:**
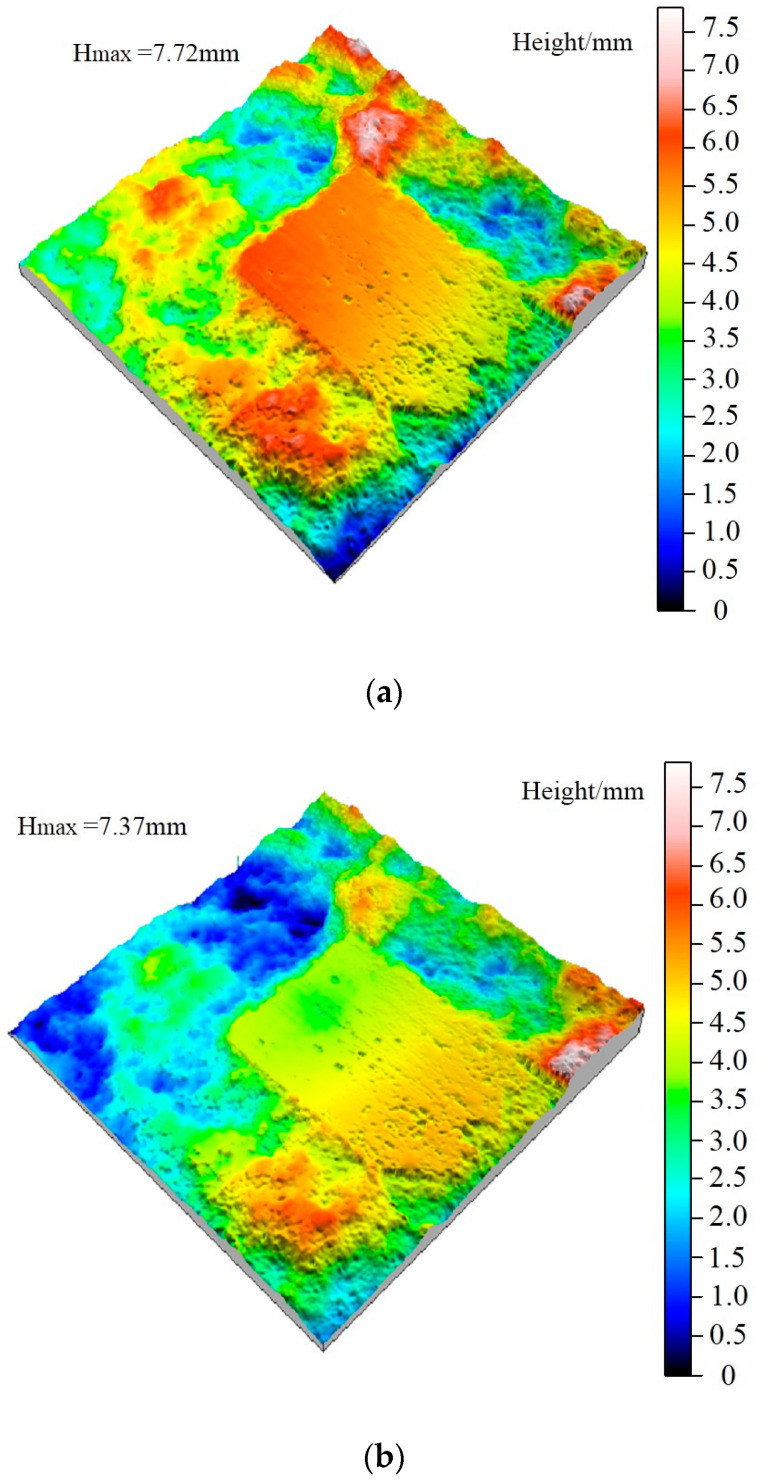

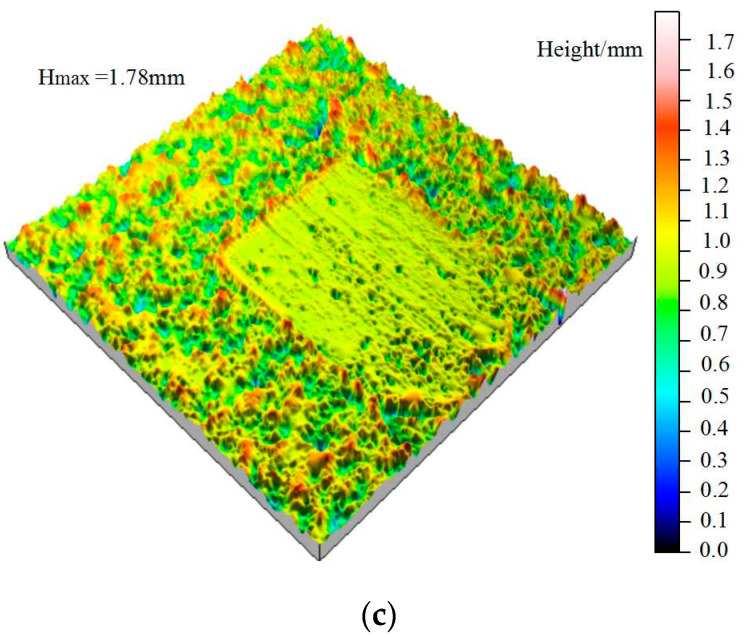
The three-dimensional topography of the shear surface before and after Gaussian filtering of the unfreeze-thaw jointed rock specimen. (**a**) The original shear surface after the first shear experiment. (**b**) Joint waviness surface after Gaussian filtering. (**c**) Joint roughness surface after Gaussian filtering.

In order to ensure that the filter size of first-order and second-order asperities is significantly different, 2.5 mm was taken as the cut-off wavelength of the Gaussian filter. Figure 5 shows the 3D morphology of the shear surface of a typical fresh (unfrozen) rock specimen before and after Gaussian filtering. Moreover, the box dimension method and TalyMap software (TalyMap Gold 5.0, Leicester, UK) were used to conduct fractal analysis on the shear surface after Gaussian filtering. The fractal dimension of the shear surface is shown in Table 1.

According to Figure 5 and Table 1, the original shear surface before Gaussian filtering is rough and has noticeable folds, while the waviness surface after Gaussian filtering is smooth and flat. Compared with the original shear surface, the fractal dimension is smaller, and folds are reduced significantly. Moreover, the undulating trend of the waviness surface is the same as that of the original shear surface, yet the height feature is slightly lower. After Gaussian filtering, the unevenness surface has no significant fluctuation trend, and the surface is full of rough second-order asperities with small height variation amplitude; yet the asperity slope is steep. Meanwhile, the fractal dimension increases significantly. Therefore, it can be concluded that the Gaussian filter can effectively separate the first- and second-order asperities on the shear surface. In addition, according to Table 1, the fractal dimension is related to the shear surface frequency components. The larger fractal dimension represents a higher frequency of second-order asperities on the shear surface and a more irregular shear surface. Similarly, a small fractal dimension means long wavelength and even shear surface. Additionally, the fractal dimension of the shear surface increases with the increase of freeze-thaw cycles. 

## 4. Three-Dimensional Roughness Parameters of the Shear Surface

At present, the research on the surface morphology characteristics in a complex environment is continually progressing, yet the two-dimensional roughness parameters based on contour lines begin to fail to meet the requirements. Moreover, the freeze-thaw cycle has an apparent damage and degradation effect on the first- and second-order surface asperities, which aggravates the complexity of the shear surface. Therefore, it is a prerequisite to study the influence of freeze-thaw cycles on the three-dimensional morphology of the shear surface by selecting appropriate evaluation parameters. According to the roughness coefficient ratio *S* proposed by Belem et al. [19], it can be used as a three-dimensional roughness parameter to evaluate the surface roughness. In addition, based on the difference value of *S* before and after shearing, the parameter representing the degree of shear surface damage, namely, shear surface wear degree *D_s_*, was proposed as the evaluation parameter to analyze the impact of freeze-thaw cycles on the surface morphology.

### 4.1. Roughness Coefficient Ratio S

**Figure 6:**
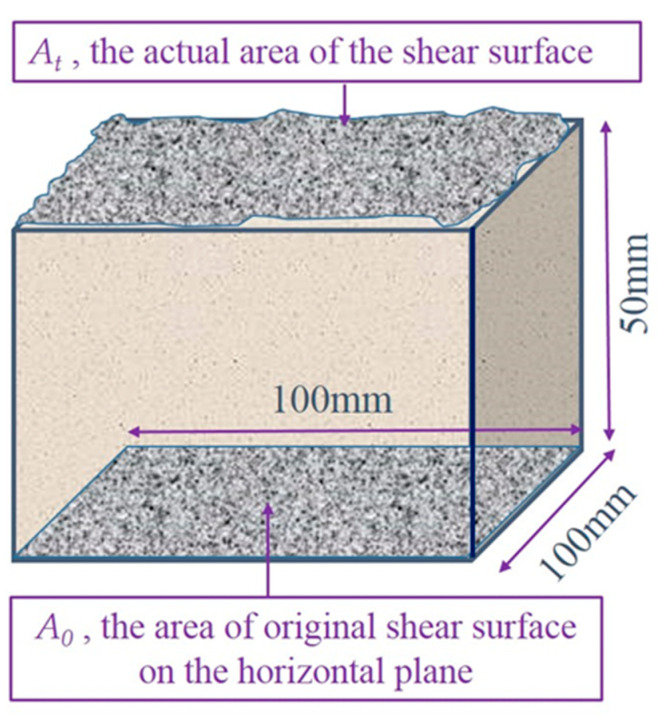
The Schematic diagram of *A_t_* and *A*_0_ in joint shear surface.

The ratio of shear surface roughness coefficient *S* represents the roughness of the shear surface relative to the horizontal surface, which is used to represent the complicated situation of the shear surface. The expression method of *S* is the incremental ratio of actual shear surface area:

(2) $$S=\frac{A_{t}-A_{0}}{A_{0}}$$

where *A_t_* is the actual area of the shear surface, and *A*_0_ is the projected area of the original shear surface on the horizontal plane. The schematic diagram of *A_t_* and *A*_0_ is shown in Figure 6.

The increase of the roughness coefficient *S* indicates an increase in the complexity of the shear surface morphology. Similar to the expression of *S*, and the roughness coefficient ratios of the (large-scale) waviness surface and the (small-scale) unevenness surface can be expressed as $S_{w}$ and $S_{r}$:

(3) $$S_{w}=\frac{A_{t}^{w}-A_{0}}{A_{0}}$$

(4) $$S_{r}=\frac{A_{t}^{r}-A_{0}}{A_{0}}$$

where $A_{t}^{w}$ is the actual area of the filtered large-scale waviness surface, and $A_{t}^{r}$ is the actual area of the filtered small-scale unevenness surface.

Moreover, according to the fractal theory [29], the expression of *A_t_* is:

(5) $$A_{t}=A_{0}\varepsilon^{2-D_{A}}$$

where ε is the reference unit code ruler of the measuring surface, and $D_{A}$ is the fractal dimension of the shear failure surface of the jointed rock specimen. Since the scanner’s accuracy is 0.01 mm, ε is defined as the equilateral triangle area with the side length of 0.01 mm, ε = 4.332 × 10^−5^ mm^2^.

Therefore, according to the fractal dimension in different states and combining Equations (2)–(5), *S*, *S_w_*, and *S_r_* can be obtained.

### 4.2. Shear Surface Degree of Wear D_s_

For the wear condition of a shear surface morphology, Homand et al. [18] proposed that wear parameters are more suitable for representing the cumulative wear degree of the shear surface in the cyclic shearing process. In order to accurately describe the damage to the shear surface caused by shearing, we set the ratio $D_{s}$ of the roughness coefficient ratio after each shearing to the decrease of the roughness coefficient ratio before shearing as a new parameter that characterizes the degree of the shear surface damage, which is the surface wear degree $D_{s}$:

(6) $$D_{s}=\frac{S^{\prime }-S}{S^{\prime }}$$

where *S* is the current shear surface roughness coefficient ratio, and $S^{\prime }$ is the shear surface roughness coefficient ratio before each shear test. 

In combination with Equations (3) and (6), it can be deduced that:

(7) $$D_{s}=\frac{A_{t}^{\prime }-A_{t}^{s}}{A_{t}^{\prime }-A_{0}}=\frac{A_{d}}{A_{t}^{\prime }-A_{0}}$$

where $A_{t}^{\prime }$ and $A_{t}^{s}$ are the actual areas of shear surfaces before and after each shear test, and $A_{d}$ is the wear area of joints in this shear test.

$D_{s}$ represents the degree of wear on the shear surface in the shearing process. If $D_{s}=0$, it indicates that the shear surface is not worn during the shearing process, and the shear does not cause the degradation to the shear surface roughness. If $D_{s}=1$, it indicates that the surface damage is the most serious in the shearing process, and the asperities are entirely smoothed. The wear of waviness and roughness surfaces is expressed in terms of $D_{w}$ and $D_{r}$ respectively. 

Similarly, $D_{w}$ and $D_{r}$ can be used to represent the wear degree of the large-scale waviness surface and the small-scale unevenness surface. The expressions of $D_{w}$ and $D_{r}$ are Equations (8) and (9), respectively:

(8) $$D_{w}=\frac{S_{w}^{\prime }-S_{w}}{S_{w}^{\prime }}$$

(9) $$D_{r}=\frac{S_{r}^{\prime }-S_{r}}{S_{r}^{\prime }}$$

where $S_{w}^{\prime }$ is the roughness coefficient ratio of the large-scale waviness surface before this shear test, and $S_{r}^{\prime }$ is the roughness coefficient ratio of the small-scale unevenness surface before the shear testing.

In combination with Equations (3) and (4) and Equations (8) and (9), it can be obtained that:

(10) $$D_{w}=\frac{A_{d}^{w}}{A_{t}^{w^{\prime }}-A_{0}}$$

(11) $$D_{r}=\frac{A_{d}^{r}}{A_{t}^{r^{\prime }}-A_{0}}$$

where $A_{t}^{w^{\prime }}$ and $A_{t}^{r^{\prime }}$ are the areas of the large-scale waviness surface and the small-scale unevenness surface before the shear test and $A_{d}^{w}$ and $A_{d}^{r}$ are the areas of wear of the (large-scale) waviness surface and the (small-scale) unevenness surface during the shear test.

## 5. Analysis of Experimental Results

### 5.1. Analysis of Shear Stress—Displacement Curves

**Figure 7:**
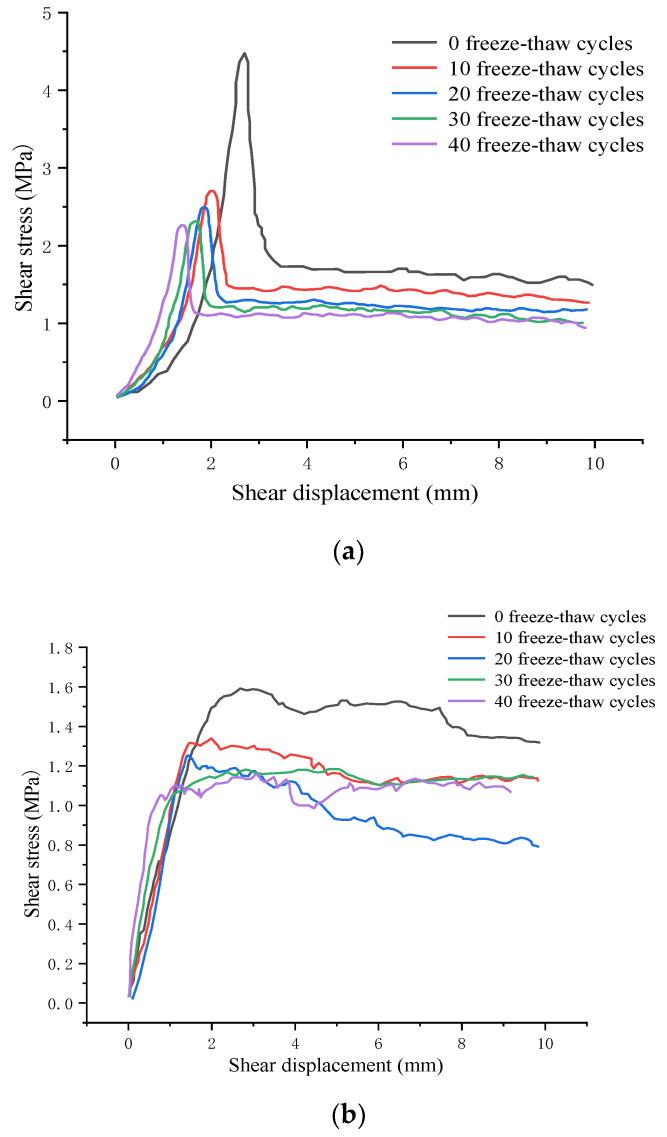

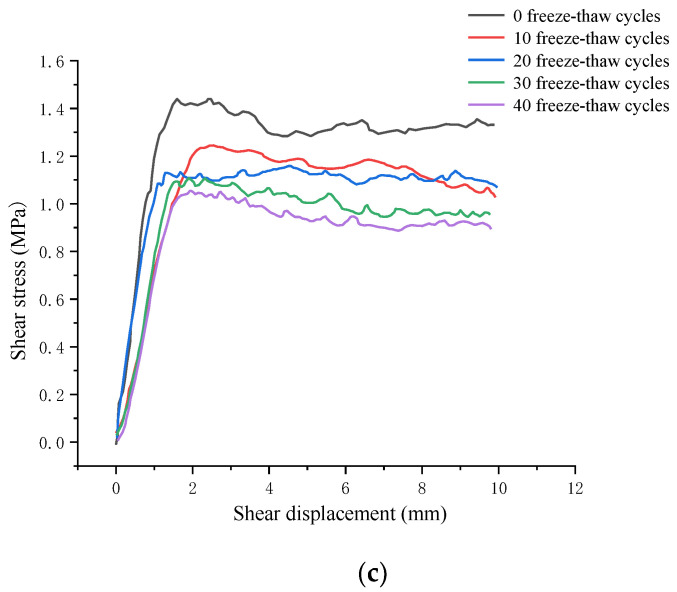
Shear stress-displacement curve of the freeze-thaw jointed rock specimen. (**a**) The first shear experiment. (**b**) The second shear experiment. (**c**) The third shear experiment.

Figure 7 shows the shear stress-displacement curve of jointed rock specimens after the freeze-thaw cycles. The experimental data with the normal stress of 1 MPa were compared and analyzed. According to Figure 7a, in the first shear experiment, the shear stress-displacement curve shows a prominent peak stage and residual stage, and the shear strength gradually decreases with the accumulation of freeze-thaw cycles. Similarly, the residual strength first decreases significantly and then decreases slowly with the increase of freeze-thaw cycles, which can be attributed to the fact that the rock mass degradation mainly occurs in the early stage of freeze-thaw cycles. After the shear failure, there is no pronounced strength drop in the second and third shear tests, as the shear strength is now provided by the friction between the upper and lower rock blocks. 

From Figure 7b,c, the joint asperities are occluding with each other, providing more excellent resistance, and the shear stress increases. In addition, when the shear stress increases to a specific value, the tip of the second-order asperities with lower strength will be cut off, leading to a decrease in shear stress. Additionally, when the joints cross the remaining second-order asperities again, the re-generated occlusion leads to the shear stress rise again until the second-order asperities are entirely cut off and flattened from the bottom. Moreover, the shear stress-displacement curve tends to be gentle because many second-order asperities have been cut and smoothed out during the first two shearing processes. Furthermore, the freeze-thaw effect causes relatively significant damage to the shear surface, and the number of first-order asperities with shear failure decreases, leading to a stable change in shear stress. 

**Figure 8:**
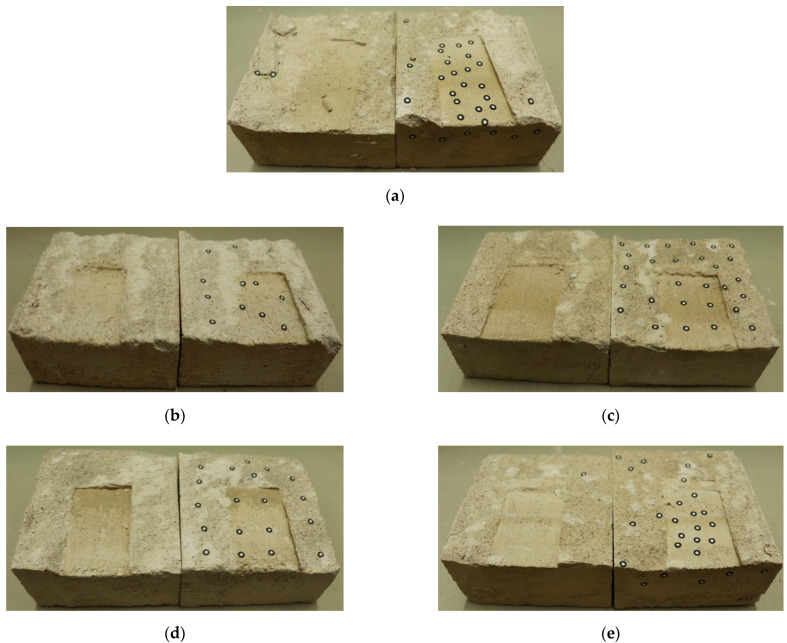
Shear surface under freeze-thaw cycles after the second shear test. (**a**) Zero freeze-thaw cycles. (**b**) Ten freeze-thaw cycles. (**c**) Twenty freeze-thaw cycles. (**d**) Thirty freeze-thaw cycles. (**e**) Forty freeze-thaw cycles.

The shear surface after the second shear test with different freeze-thaw cycles is shown in Figure 8. According to Figure 8a, the macroscopic undulations and irregular lines on the shear surface without freeze-thaw cycles are significant, while the surface wear degree is relatively less obvious. By comparing with Figure 8b–e, it can be concluded that, with the increase of freeze-thaw cycles, the shear surface gradually tends to be smooth, the surface macro-undulating gradually decreases, and the wear degree also tends to be significant. Especially after 40 freeze-thaw cycles, the shear surface is almost entirely smooth. This is due to the increase of freeze-thaw cycles, jointed rock specimens are significantly damaged by freeze-thaw, and surface undulations are more likely to be damaged. After shearing and rubbing, the surface becomes smoother, the undulations that can provide friction resistance during shearing become less, and the degree of shear surface occlusion decreases, which inevitably leads to the reduction of the peak shear strength.

**Figure 9:**
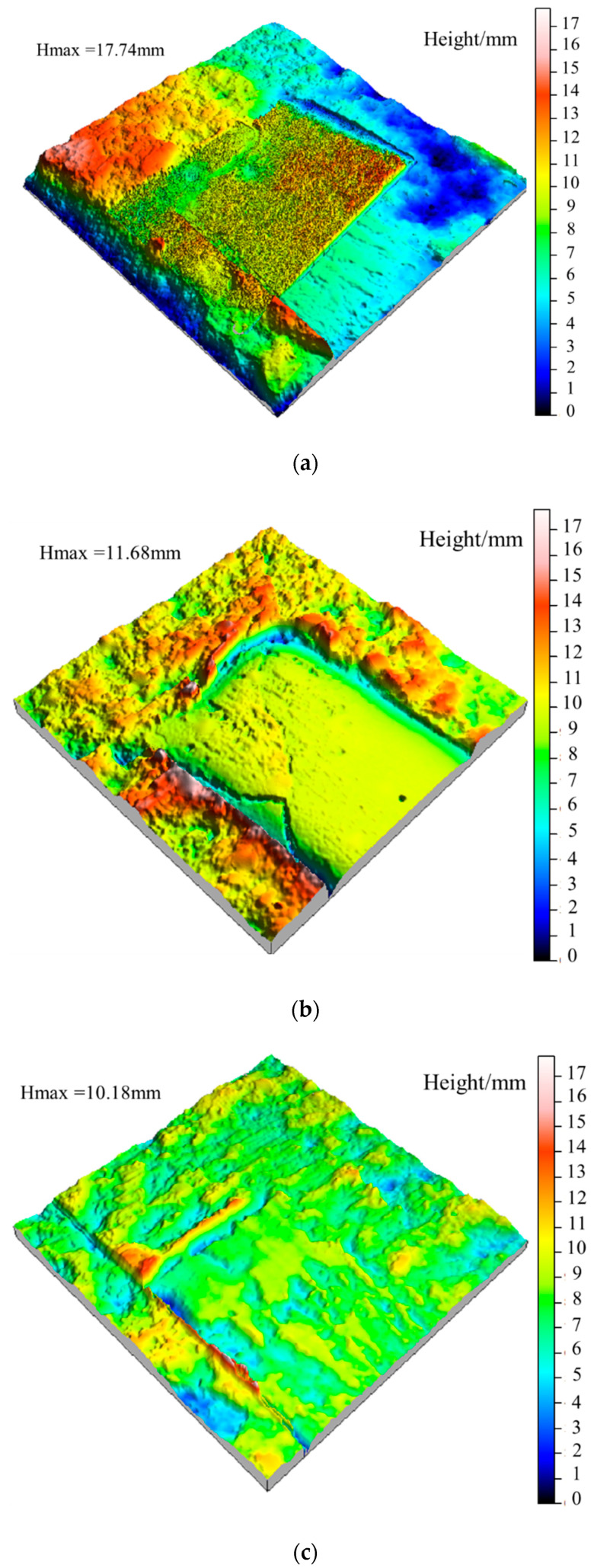

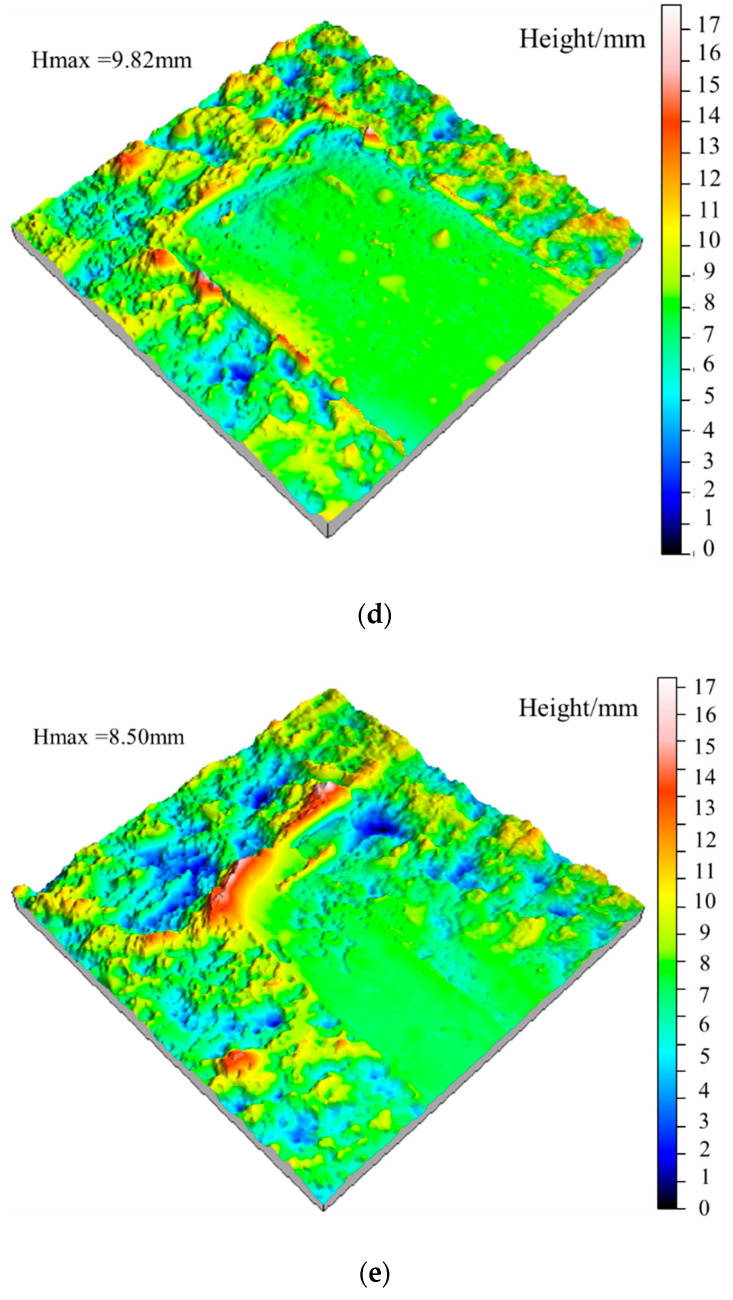
Original 3D morphology of the shear surface under freeze-thaw cycles after the second shear test. (**a**) Zero freeze-thaw cycles. (**b**) Ten freeze-thaw cycles. (**c**) Twenty freeze-thaw cycles. (**d**) Thirty freeze-thaw cycles. (**e**) Forty freeze-thaw cycles.

The original morphology of the shear surface after freeze-thaw is shown in Figure 8. According to Figure 9a, the relief height of the shear surface is 17.74 mm, which is significantly higher than that after freeze-thaw cycles. Combined with Figure 9b, it can be concluded that the shear strength of the undulating body on the shear surface under unfreeze-thaw is more significant, resulting in the peak strength being significantly higher than that of the jointed rock specimens after freeze-thaw cycles.

**Figure 10:**
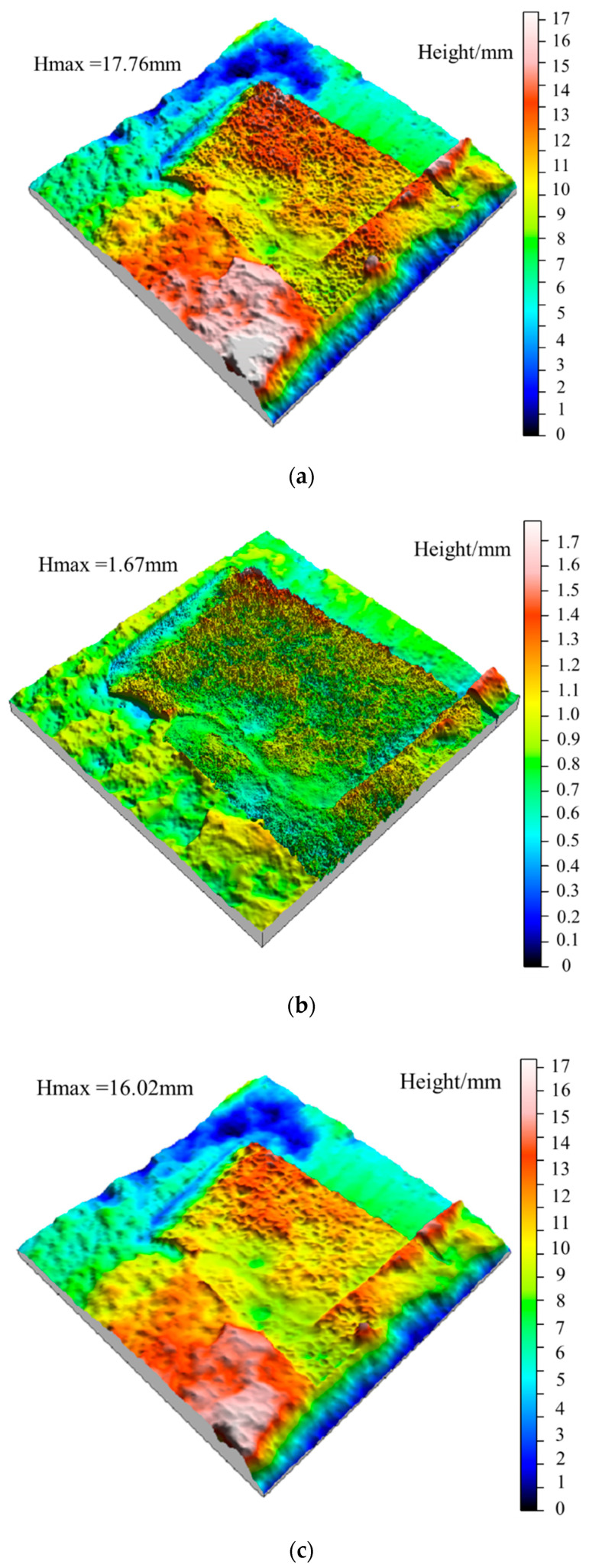

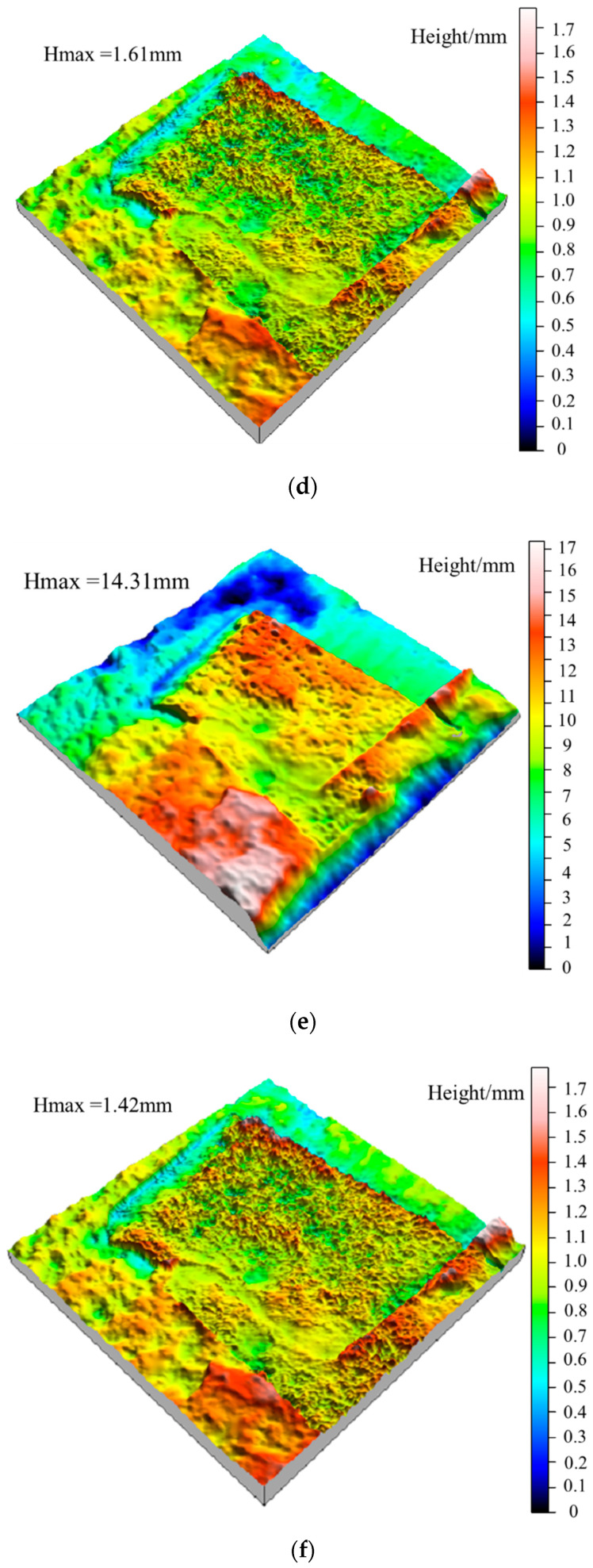
Three-dimensional topography of surface waviness and unevenness after shearing of the unfrozen specimen. (**a**) The waviness morphology after the first shear experiment. (**b**) The roughness morphology after the first shear experiment. (**c**) The waviness morphology after the second shear experiment. (**d**) The roughness morphology after the second shear experiment. (**e**) The waviness morphology after the third shear experiment. (**f**) The roughness morphology after the third shear experiment.

From Figure 10b–e, the undulation height of the shear surface decreases slightly with the increase of freeze-thaw cycles, leading to a significant reduction in the shear strength of the jointed rock specimen in the second shear test, as shown in Figure 8b. 

Based on Equations (2) and (6), the *S* values of the shear surface after 0~40 freeze-thaw cycles are 40.39%, 35.70%, 32.99%, 30.10% and 27.17%, while the *D_s_* values are 7.62%, 12.32%, 9.43%, 6.77% and 5.13%. Therefore, it can be concluded that as the freeze-thaw cycles increase, the roughness coefficient ratio *S* of the shear surface becomes lower, and the decreased amplitude of *S* is the largest in the early stage of freeze-thaw cycles. Meanwhile, the wear degree $D_{s}$ of the shear surface in the early stage of freeze-thaw cycles is greater than that under the un-freeze-thaw cycles, while the wear degree of the shear surface gradually decreases in the late stage of freeze-thaw cycles, the above variation is consistent with the law reflected in Figure 8c,d.

Therefore, the damage and deterioration of the jointed rock specimen caused by freeze-thaw are mainly concentrated in the early stage of freeze-thaw cycles, where *S* decreases the most and $D_{s}$ increases the most, leading to a significant decrease in shear strength and the height of the undulating body on the shear surface. Moreover, the more freeze-thaw cycles accumulated, the lower height of the undulating body of the shear surface, and the lower the shear strength. Similarly, the lower the roughness coefficient ratio *S* of the shear surface, the higher degree of wear *D_s_* of the shear surface.

### 5.2. Variation of Waviness and Unevenness Surface Morphologies 

**Figure 11:**
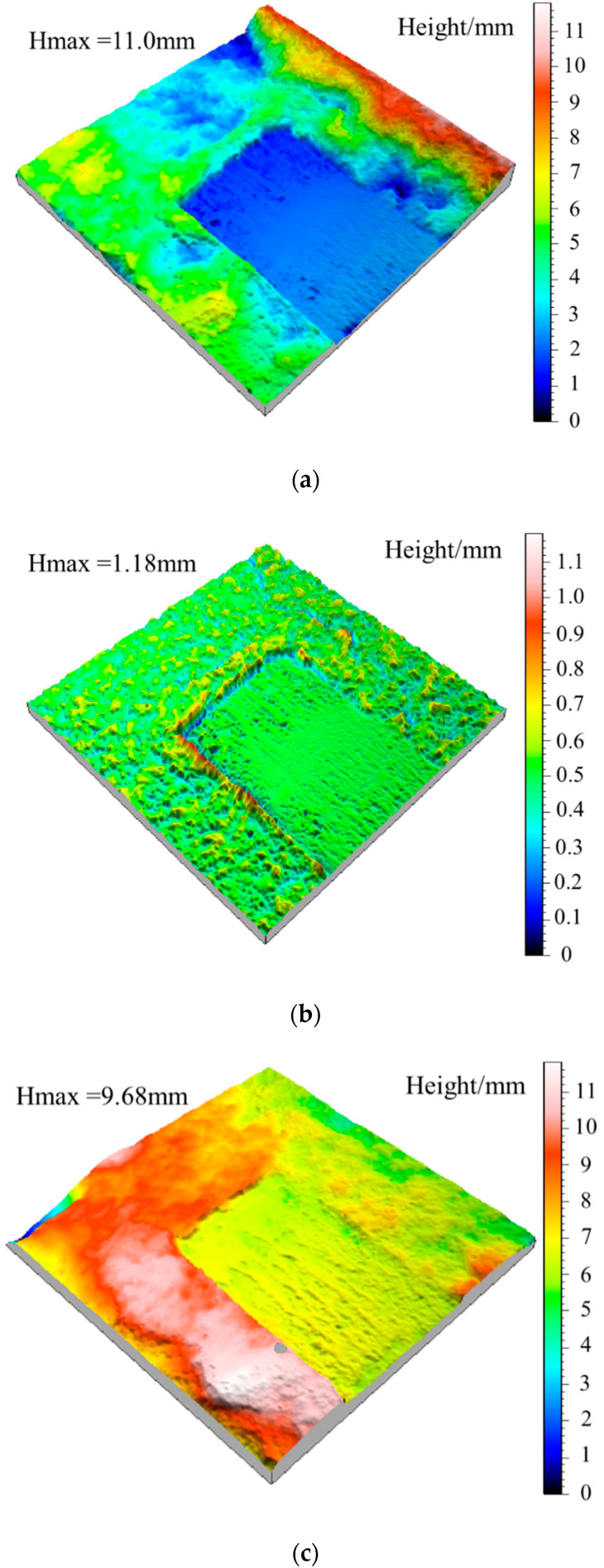

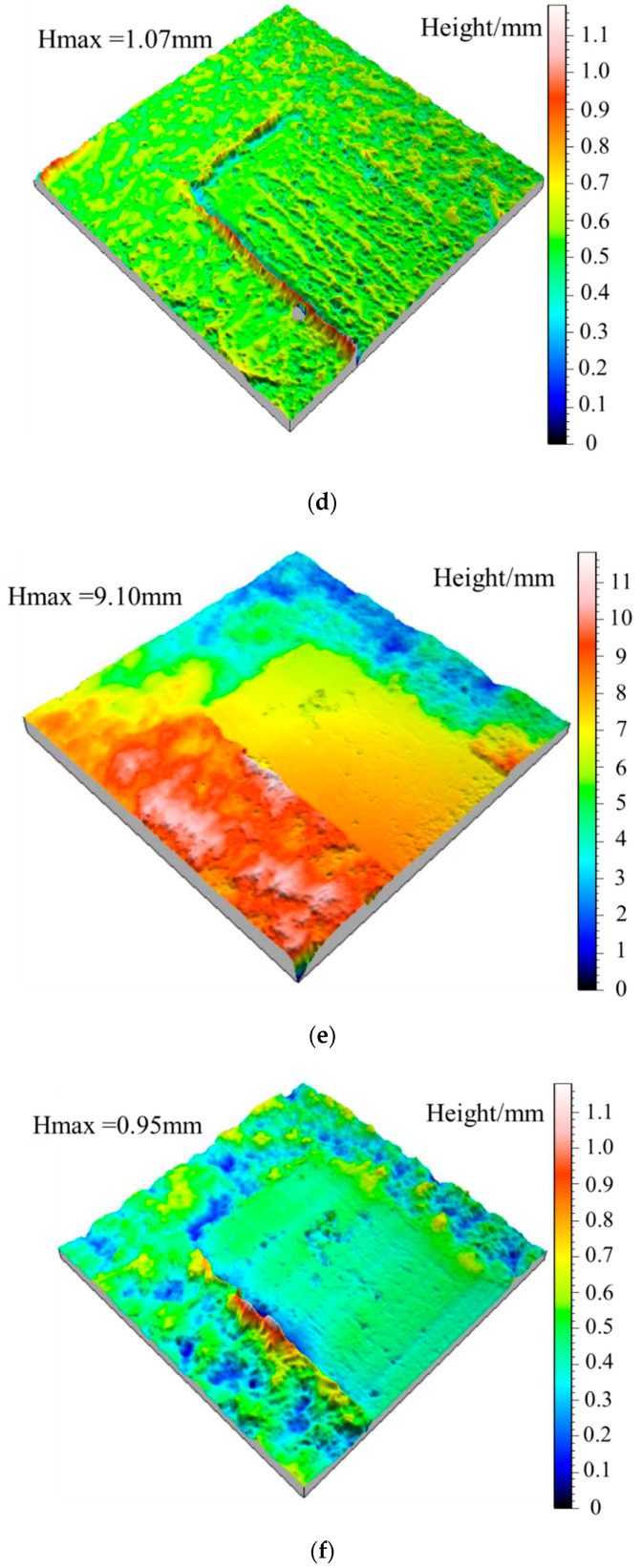

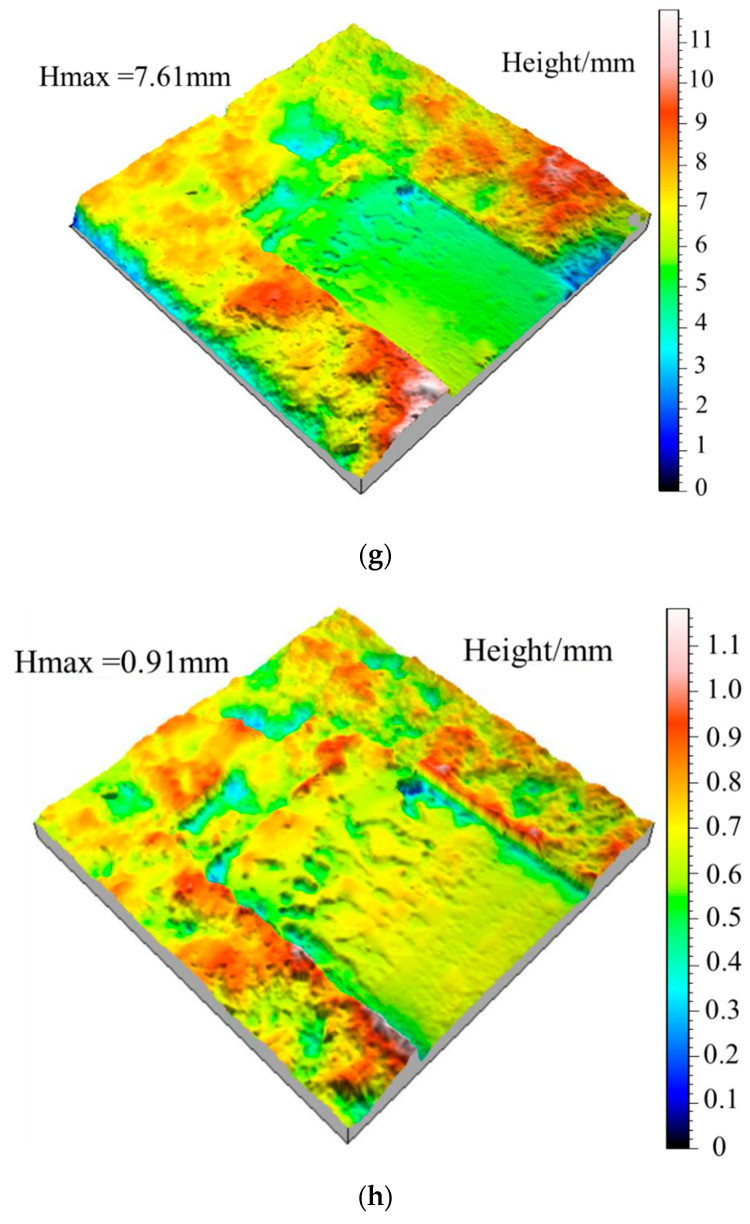
Three-dimensional topography of waviness and unevenness of jointed rock specimen after the second shear test under freeze-thaw cycles. (**a**) The waviness morphology of specimen after ten freeze-thaw cycles. (**b**) The unevenness morphology of specimen after ten freeze-thaw cycles. (**c**) The waviness morphology of specimen after 20 freeze-thaw cycles. (**d**) The unevenness morphology of specimen after 20 freeze-thaw cycles. (**e**) The waviness morphology of specimen after 30 freeze-thaw cycles. (**f**) The unevenness morphology of specimen for 30 freeze-thaw cycles. (**g**) The waviness morphology of specimen after 40 freeze-thaw cycles. (**h**) The unevenness morphology of specimen after 40 freeze-thaw cycles.

According to the Gaussian filtering method, the fresh jointed rock specimens and the jointed rock specimens under the freezing-thawing cycles were investigated, where the (large-scale) waviness surface and (small-scale) unevenness surface after shear tests were extracted, as shown in Figure 10 and Figure 11. According to Figure 10a,b, for fresh jointed rock specimens, most tips of the second-order asperities are cut off during the first shear test, and only a few asperities tips survive on both sides, and the waviness and unevenness degrees of the shear surface decreased. Meanwhile, in Figure 10c,d, the large-scale waviness surface height decreases significantly after the second shear test, the small-scale unevenness surface tends to be smooth, and most of the second-order asperities are worn and sheared off, which are consistent with the variation of the shear curve in Figure 10. Additionally, from Figure 10e,f it can be obtained that the small-scale unevenness surface height still decreases after the third shear test, yet the decrease rate is becoming lower and lower; this finding also conforms to the above variation of the shear curve. After the second shear test, the topography of the large-scale waviness surface changes significantly in the highly protruding part, and after the third shear, a prominent smooth area in the middle also emerges, and the overall wave height decreases significantly. 

Besides, the height of the large-scale waviness surface and the small-scale unevenness surface after freeze-thaw cycles decreases significantly according to Figure 11, indicating that the freeze-thaw cycle has a tremendous deteriorating effect on the surface roughness. It can be obtained from the analysis of Figure 11a,b, after ten freeze-thaw cycles, that the small-scale unevenness surface height decreases most significantly. Due to freeze-thaw damage, the large-scale waviness surface tends to be smoother than that of unfrozen rocks, and there are a few higher asperities on both sides of the joint, which mainly results from the fractured shear surface produced by the frost heaving process. Combined with the shear curve in Figure 7b, it is found that the shear peak strength and residual strength after freeze-thaw cycles are significantly lower than unfrozen rock, which is consistent with the characteristics of shear surface *S* and *D_s_*.

From Figure 11c,d, after 20 freeze-thaw cycles the height of the large-scale waviness surface and small-scale unevenness surface continued to decrease, yet the decreasing rate is reduced significantly, and the number of second-order asperities decreases significantly. Compared with the ten freeze-thaw cycles, the height of the undulating body on the shear surface decreased slightly, indicating that the variation amplitude of the roughness and wear degree of the shear surface decreased, which was consistent with the variation of the shear curve in Figure 7b. Finally, according to Figure 11e–h, after 30 freeze-thaw cycles the surface roughness does not fluctuate obviously, and the waviness shows a uniform distribution of overall height and presents a smooth state. Subsequently, after 40 freeze-thaw cycles the overall height of the waviness surface decreases slightly, and there is a small area prominent failure area at the joint edge. However, the overall large-scale waviness surface and small-scale unevenness surface height of the shear surface do not fluctuate much, indicating that the damage degree of the shear surface in the later stage of freeze-thaw cycles has tended to be stable, leading to the shear strength in this case very closely. The shear curves in Figure 7b,c are consistent with the above characteristics.

Therefore, the freeze-thaw cycle has a significant deteriorating effect on the roughness of the shear surface, and the degradation is mainly concentrated in the early stage of the freeze-thaw cycle. Furthermore, the surface roughness varies under different shear states after freeze-thaw treatment, which affects the shear properties directly. 

### 5.3. Variation of Three-Dimensional Roughness Parameters 

After each shear test, the shear surface region and three-dimensional roughness parameters were analyzed. According to Table 2, the actual surface area with different freeze-thaw cycles decreased significantly after the second shear test. The roughness coefficient ratio *S* also gradually decreases, indicating that the surface asperities are smoothed and cut off during the shearing process. Moreover, the shear surface after shearing becomes smoother and flatter, resulting in a decrease in the value of $A_{t}$, and with the accumulation of freeze-thaw cycles, the area loss of the shear surface becomes more serious.

Simultaneously, during each shear process, the jointed rock specimen corresponding to ten cycles of freeze-thaw has the largest surface area loss, indicating that the shear surface suffers the greatest degree of deterioration damage at the early stage of the freeze-thaw cycle, so the wear is the most severe. After three times of shear action, the value $A_{t}$ for fresh jointed rock specimens decreased by 15.62%, and *S* decreased by 32.50%. The value of $A_{t}$ for the rock with 10, 20, 30, and 40 cycles of freeze-thaw decreases by 18.04%, 19.87%, 21.07%, and 21.86%, respectively, the value of *S* decreases by 38.04%, 39.62%, 40.55%, and 42.13%, respectively. Therefore, the results show that the joint damage is more serious in the shearing process under the action of freeze-thaw cycles, the degradation of surface roughness is more obvious, and the degradation is aggravated with the increase of freeze-thaw cycles.

**Figure 12:**
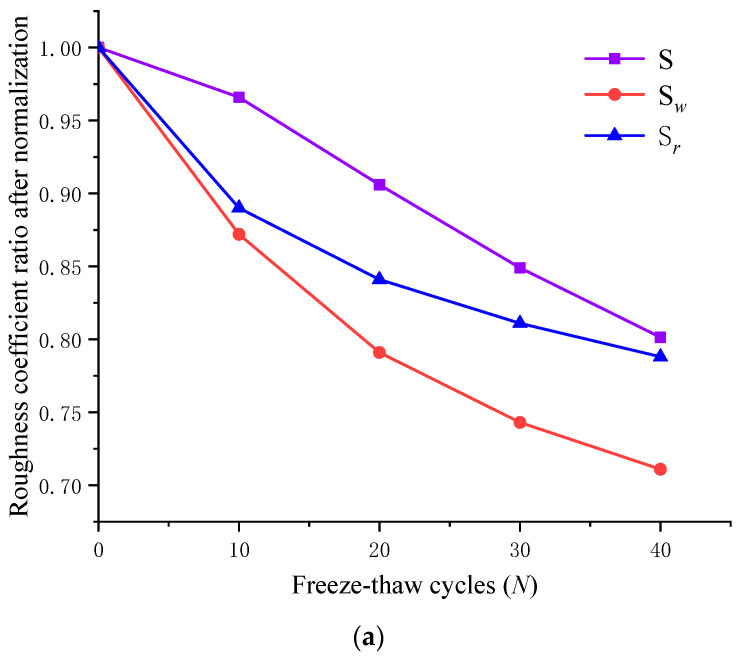

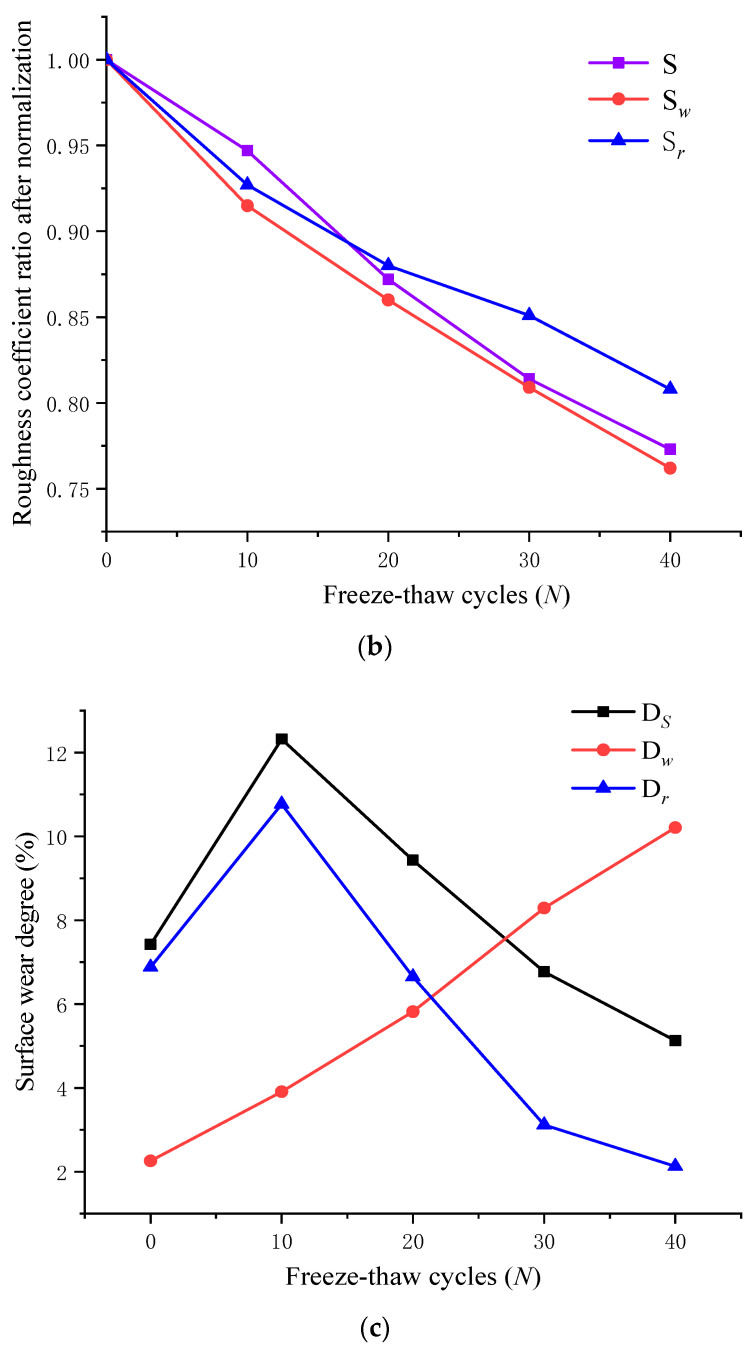

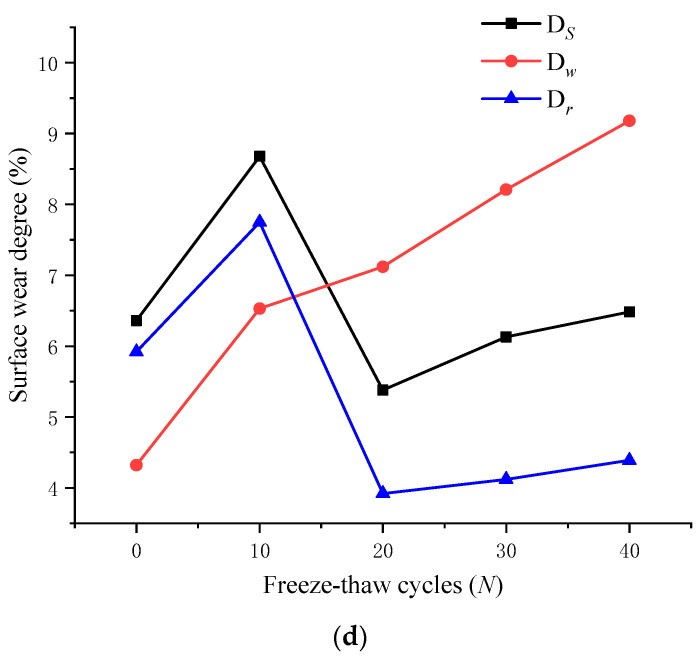
Variation of three-dimensional roughness parameters of the shear surface under freeze-thaw cycles. (**a**) Surface roughness coefficient after the second shear test. (**b**) Surface roughness coefficient after the third shear test. (**c**) Surface wear degree of the jointed rock specimens after the second shear test. (**d**) Surface wear degree of the jointed rock specimens after the third shear test.

Similar to Table 2, the area before and after the shear failure of the large-scale waviness surface and small-scale unevenness surface can be obtained, respectively. As a result, by substituting Equations (2)–(4) and (7)–(9), we can calculate the roughness coefficient ratio between the large-scale waviness surface, the small-scale unevenness surface, and the wear degree of the shear surface. Based on the roughness coefficient ratio of fresh shear surface, the roughness coefficient ratios of freeze-thaw jointed rocks were normalized. Figure 12 shows the variation curves of the three-dimensional roughness parameter of the jointed rocks after shear under different freeze-thaw cycles. It shows that: 

(1) After freeze-thaw cycles, *S*, $S_{w}$ and $S_{r}$ of the shear surface show an overall downward trend, indicating that both the original shear surface and the Gaussian filtered large-scale waviness surface and the small-scale unevenness surface show an overall decrease in surface roughness after freeze-thaw cycles. Due to freeze-thaw degradation, the asperity is more prone to wear in the shearing process, which leads to the decrease of the shear surface undulation and the flattening of surface morphology. The $S_{w}$ curve of the waviness surface gradually becomes steeper, while the $S_{r}$ curve of the uneven surface tends to be gentle. This indicates that with the increase of freeze-thaw cycles the large-scale waviness surface and the small-scale unevenness surface show an accelerated downward trend, while the roughness of the small-scale unevenness surface shows a moderate downward trend. 

(2) The $D_{w}$ of the large-scale waviness surface shows an increasing trend on the whole. For the small-scale unevenness surface, the $D_{r}$ after 10 freeze-thaw cycles is significantly higher than that after 20, 30, and 40 freeze-thaw cycles, while the variation of $D_{s}$ remains similar. Based on the analysis of Figure 8, Figure 9 and Figure 10, it is concluded that the freeze-thaw cycle causes significant damage to the first-order and second-order asperities on the shear surface, yet the damage degree is different, leading to different failure modes under shear action. Therefore, the above factors are the main reasons for the inconsistent variation of the large-scale waviness surface and the small-scale unevenness surface. For the small-scale unevenness surface, the strength of the second-order asperity is weak, and most of them are smoothed and cut in the shear process after 10 freeze-thaw cycles so that the $D_{r}$ decline amplitude decreases gradually after 20 freeze-thaw cycles. With the increase of freeze-thaw cycles, the damage to the surface asperity intensifies, leading to the constant increase of the large-scale waviness surface wear. 

Additionally, according to Figure 12c,d, it is concluded that the curve of $D_{r}$ and $D_{w}$ have an intersection point, which means that when the freeze-thaw is more significant than a certain cycle number, the large-scale waviness surface wear degree begins to exceed the small-scale unevenness surface wear degree. Moreover, the number of cycles corresponding to the second shear test is about 20, corresponding to the third shear test is about 13 cycles.

(3) When the freeze-thaw cycle is repeated ten times, *D_s_* is closer to *D_r_*. Subsequently, with the continuous increase of the freeze-thaw cycle, the difference between $D_{s}$ and $D_{r}$ increases gradually, while the difference between $D_{s}$ and $D_{w}$ decreases gradually. Simultaneously, *S,* $S_{w}$, and $S_{r}$ also have the same variation law. Therefore, it can be concluded that in the initial stage of the freeze-thaw cycle, the roughness of the shear surface is primarily dominated by the small-scale unevenness surface, so the wear of second-order asperity is the main factor of shear surface wear. Then, with the increase of freeze-thaw cycles, the damage of the first-order asperity intensifies, so the large-scale waviness surface mainly controls the variation of shear surface morphology. 

## 6. Conclusions

A large number of studies have shown that freeze-thaw cycles will reduce the mechanical strength of jointed rock mass, and its microscopic characteristics influence the morphological characteristics of the potential joint-related shear surface. However, the morphology of the shear surface and its evolution process under freeze-thaw cycles have not been reported. In this paper, freeze-thaw cycles experiments are carried out on several groups of joints of different sizes at different temperatures. The internal relationship between the surface morphology and shear strength under freeze-thaw cycles is studied based on the Gaussian filter analysis method. The results are as follows:

(1) After the freeze-thaw cycle, the actual shear surface area decreases more significantly than fresh jointed rocks during the shear test, and the roughness coefficient ratio S also decreases significantly. Meanwhile, the roughness of the waviness and roughness surfaces decreases significantly with the increase of freeze-thaw cycles.
(2) Surface wear degree *D* can be used to characterize the degree of shear surface damage. With the increase of freeze-thaw cycles, the $D_{w}$ of the waviness surface and the $D_{r}$ of the unevenness surface change differently, which is related closely to the failure mode of the asperity caused by freeze-thaw damage. The failure of the first-order asperity increases with freeze-thaw cycles, leading to the gradual increase of surface wear degree *D_w_* of waviness. At the early stage of the freeze-thaw cycles, most second-order asperities are flattened during shearing, causing the uneven surface wear degree $D_{r}$ to be high first and then low. As the freeze-thaw cycles increase, the damage of the surface asperities intensifies, leading to the constant increase of the waviness surface wear degree.
(3) At the early stage of freeze-thaw cycles, the morphology variation of the shear surface and its unevenness surface after the shear test are similar. During this period, the damage of the shear surface is mainly dominated by the roughness surface, so the wear of the second-order asperities is the main factor of the shear surface wear. However, with the increase of freeze-thaw cycles, the damage degree of the first-order asperity becomes the critical factor determining the surface wear characteristics, so the waviness surface would mainly control the evolution of the shear surface morphology.

This study demonstrates the practicability of Gaussian filtering in rock mechanics. The more important aspect is that this method reveals the internal mechanism and control parameters of the shear surface wear characteristics. The results of the study reveal a novel way that the macroscopic shear strength variation can be inferred by grasping the microscopic shear surface morphology variation. In the future, related research work will be further deepened, primarily focusing on refining related theories and models, and mathematically establishing the relationship between microscopic shear surface morphology and macroscopic shear strength variation.

## Figures and Tables

**Table 1:** Fractal dimension of the shear surface before and after Gauss filtration in the first shear test.

Freeze-Thaw Cycles (*N*)	Original Shear Surface	Waviness Surface	Unevenness Surface
0	2.12	2.10	2.34
10	2.16	2.13	2.36
20	2.19	2.16	2.39
30	2.20	2.17	2.43
40	2.26	2.21	2.45

**Table 2:** Three-dimensional roughness parameters of jointed rock specimens before and after shearing.

Shear Test Times	Freeze-Thaw Cycles (*N*)	$A_{0}/{\mathrm{mm}}^{2}$	$A_{t}/{\mathrm{mm}}^{2}$	$A_{r}/{\mathrm{mm}}^{2}$	$A_{w}/{\mathrm{mm}}^{2}$	*S*/%	$D_{S}/\%$
2	0	10,000	14,039.5	11,880.1	10,633.9	40.39	7.62
	10		13,570.4	11,681.3	10,611.2	35.70	12.32
	20		13,299.2	11,570.6	10,577.3	32.99	9.43
	30		13,010.6	11,520.4	10,530.6	30.10	6.77
	40		12,717.9	11,249.3	10,321.4	27.17	5.13
3	0	10,000	12,650.3	11,200.4	10,298.7	26.50	6.46
	10		12,421.7	11,110.6	10,281.3	24.21	8.68
	20		12,292.3	11,071.5	10,263.4	22.92	5.38
	30		12,142.6	11,029.8	10,222.6	21.43	6.13
	40		11,932.7	10,824.5	10,018.8	19.33	4.84

Note: $A_{w}$ and $A_{r}$ are the areas of the waviness and unevenness surfaces after Gaussian filtering.

## Data Availability

Some or all data, models, or code that support the findings of this study are available from the corresponding author upon reasonable request.
